# Extreme geographic misalignment of healthcare resources and HIV treatment deserts in Malawi

**DOI:** 10.1038/s41591-025-03561-6

**Published:** 2025-03-14

**Authors:** Joan Ponce, Justin T. Okano, Andrea Low, Luckson Dullie, Wongani Mzumara, Sally Blower

**Affiliations:** 1 Center for Biomedical Modeling, Semel Institute for Neuroscience and Human Behavior, David Geffen School of Medicine, University of California Los Angeles, Los Angeles, CA USA; 2 ICAP at Columbia University, Mailman School of Public Health, Columbia University, New York, NY USA; 3 Partners In Health/Abwenzi Pa Za Umoyo, Neno, Malawi; 4 Department of HIV and AIDS, Ministry of Health Malawi, Lilongwe, Malawi; 5 Present Address: School of Mathematical and Statistical Sciences, Arizona State University, Tempe, AZ USA

**Keywords:** HIV infections, Epidemiology

## Abstract

The Joint United Nations Programme on HIV and AIDS has proposed that human rights should be at the center of efforts to end the HIV pandemic and achieving equity in access to antiretroviral therapy (ART) and HIV healthcare is essential. Here we present a geospatial and geostatistical modeling framework for conducting, at the national level, an equity evaluation of access to ART. We apply our framework to Malawi, where HIV prevalence is ~9%. Access depends upon the number of available healthcare facilities (HCFs), the travel times needed to reach these HCFs, the mode of transportation used (walking, biking, driving) and the supply-to-demand ratio for ART at the HCFs. We find extreme inequities in access to ART. Access maps show striking geographic patterns, revealing clusters of communities with very low or high levels of access. We discover that an extreme geographic misalignment of healthcare resources with respect to need has generated a new type of medical desert: an HIV treatment desert. Around 23% of people living with HIV reside in deserts where they have to walk up to 3 h to reach HCFs; in 2020, these HCFs only received 3% of the national supply of ART. We recommend strategies for shrinking deserts; if not implemented, deserts will grow in size and number.

## Abstract (web summary)

Geospatial modeling analyses in Malawi identified HIV treatment deserts, clusters of communities with either very low or high levels of access to HIV treatment services, reflecting extreme geographic misalignment of healthcare resource allocation.

## Main

The HIV pandemic is centered in sub-Saharan Africa (SSA), where ~25.5 million people live with HIV infection^1^. In 2024, the Joint United Nations Programme on HIV and AIDS (UNAIDS) proposed an approach that puts human rights at the center of efforts to end the pandemic by 2030: this is referred to as ‘Take the Rights Path to End AIDS’^2^. The underlying foundation of this approach is to eliminate inequalities in access to antiretroviral therapy (ART) and HIV healthcare. One important inequality is geographic. It is essential to eliminate geographic inequalities in access, because, if severe, these inequalities can generate medical deserts^3^. These are areas where there is low access to healthcare resources and access is specified in terms of distance or travel time^3^. To date, many types of medical deserts have been found to exist: for example, pharmacy deserts^4^, mental healthcare deserts^5^, contraceptive deserts^6^ and vaccine deserts^7^.

Geographic inequalities in access to ART and HIV healthcare have previously been evaluated by analyzing differences in utilization rates (represented by differences in the ART coverage level) between urban and rural populations^8^; these inequalities have not been analyzed from a geospatial perspective. A 2024 report of 15 African countries based on survey data from the Population-Based HIV Impact Assessment (PHIA) Project showed that, on average, there was lower treatment coverage and worse treatment outcomes (higher AIDS-related deaths) among rural compared with urban communities^8^. In addition, these data showed that poorer compared with richer communities, men as opposed to women, and individuals younger than 25 years old were similarly disadvantaged in terms of coverage and outcomes. Although men have lower ART coverage than women, HIV incidence (and prevalence) in women is far greater than in men: in Africa, the highest incidence rate is in adolescent girls and young women^2^.

**Fig. 1:**
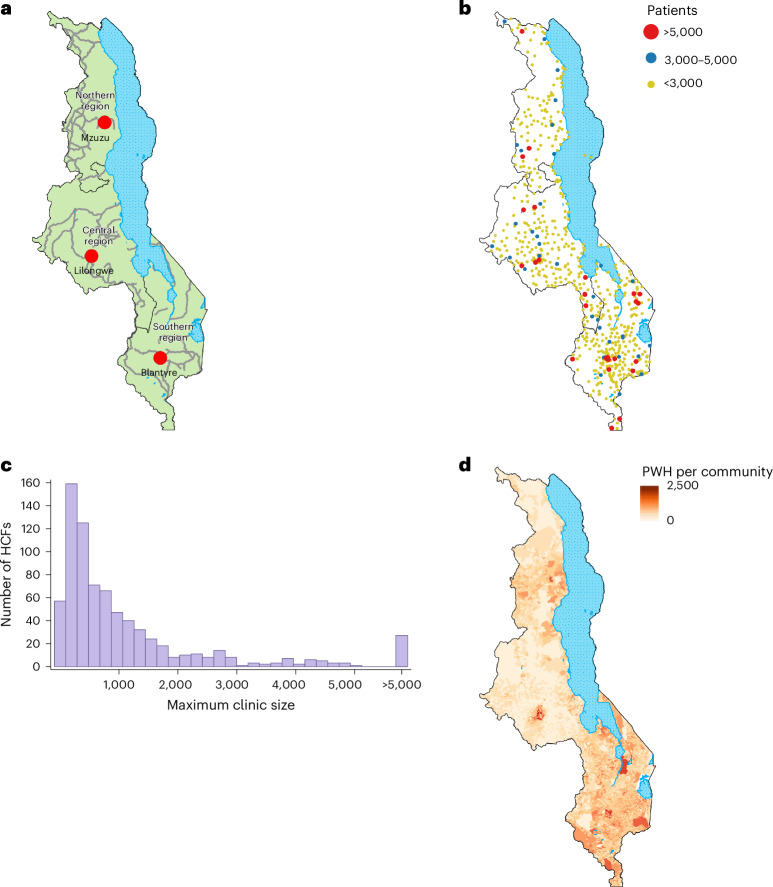
Health geographics in Malawi and the geographic distribution of people living with HIV. **a**, Map of Malawi showing regions (solid black line demarcations), cities (red circles), water bodies (dotted light blue areas), and primary and secondary roads (gray lines). **b**, Map showing the geographic location and clinic size of the 758 HCFs that provided ART in 2020: >5,000 patients (red dots), 3,000–5,000 patients (blue dots) and <3,000 patients (yellow dots). The solid lines delineate Malawi’s three regions. **c**, Histogram of clinic size in terms of the maximum quarterly number of patients provided with ART in 2020. **d**, Density of infection map showing the number of people living with HIV (PWH) in each of the 9,208 communities in Malawi.

Here we present a geospatial and geostatistical modeling framework for conducting, at the national level, an equity evaluation of access to ART: our analysis focuses on geographic inequalities. We apply our framework to Malawi (Fig. 1a) because it has one of the most severe HIV epidemics in the world: HIV prevalence was 9% in the general population in 2020–2021^9^. The country is close to UNAIDS treatment targets for 2030^10^ but still needs more people living with HIV on treatment: 14% of people living with HIV in Malawi were in need of ART in 2020–2021^11^. The aims of our study are (1) to calculate access to ART for every community in Malawi, (2) to use econometrics to evaluate the degree of inequity in access at the national level, (3) to identify geographic areas where there are gaps in health services and (4) to evaluate the geographic distribution of resources for HIV healthcare relative to need. We use our results to recommend strategies for increasing equity in access to ART and attaining UNAIDS 2030 targets using a human-rights-based approach^2^.

## Results

### Study design

To conduct our analysis, we calculated the spatial accessibility of ART for every community in Malawi. Spatial accessibility is a widely used metric in the fields of health policy, public health and health geographics; it measures the opportunity that a community has to access a specific resource^12–21^. To model the spatial accessibility of healthcare, a metric is calculated that takes into account the geographic infrastructure of the healthcare system, the time needed to reach the location where healthcare is provided (referred to as travel time) and the relationship between the supply of, and demand for, the healthcare resource being accessed. Floating catchment area (FCA) models are frequently used for calculating the spatial accessibility of many types of healthcare and identifying geographic inequalities in access^12–21^. Here we use the balanced FCA model^22^ to calculate a metric we refer to as the spatial accessibility of ART (SAA) index. Our index functions as a health service metric to measure the degree of geographic inequity in the provision of HIV healthcare and to identify gaps in services. Subsequently, we refer to ‘spatial accessibility’ as ‘accessibility’ and ‘spatial access’ as ‘access’.

Our FCA model includes the entire national HIV healthcare infrastructure and every community in Malawi. Each census unit (that is, enumeration area) is assumed to contain one community; there are 9,208 enumeration areas. In 2020, 758 healthcare facilities (HCFs) provided ART (Fig. 1b); ~100% of people living with HIV picked up their medications at these facilities^23^. The supply of ART at each HCF was defined as the maximum quarterly number of people living with HIV treated with ART in 2020; this ranged from 5 patients at a rural clinic to 25,067 patients at the Bwaila District Hospital in Lilongwe (Fig. 1c). Communities contained ~1,000 individuals (range: 2–23,967) aged 15 or older. The demand for ART in each community was defined as the total number of people living with HIV (aged 15 or older) living in that community; this ranged from 0 to 2,478 people living with HIV per community (Fig. 1d). We estimate that there were a total of 1,035,525 people living with HIV aged 15 or older in Malawi in 2020.

We calculated the value of the SAA index for each community; higher values represent greater access. The value depends upon the geographic location of the community, the geographic location of all HCFs in the catchment area surrounding the community, the travel time needed to reach each HCF, the type of transportation used and the supply-to-demand ratio for ART in the catchment area around each HCF (that is, the localized supply-to-demand ratio). Within the model, multiple communities can use the same HCF and each community can use multiple HCFs. To estimate travel times, we constructed an impedance map^24^ of Malawi: a three-dimensional representation based on topography, vegetation, rivers and other water bodies, and road networks. Precise geographic delimitation of the catchment area around HCFs is uncommon in SSA^25^. Therefore, we conducted a spatial sensitivity analysis and varied catchment size. We examined six sizes by varying the maximum one-way travel time (1 h, 2 h or 3 h) and considering two modes of transportation (walking only or a combination of motorized transportation, bicycling and walking). Most Malawians walk to access healthcare. In 2020–2021, only 2% of households owned cars or trucks, 4% owned motorbikes or scooters, and 34% owned bicycles^23^.

### Variation in access

**Fig. 2:**
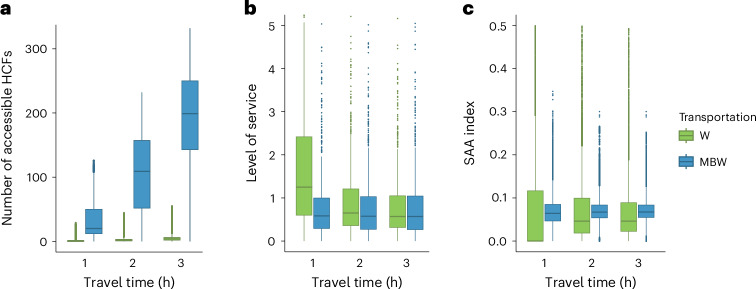
Variation in access to ART. Each set of boxplots shows six catchment sizes that are based on varying the maximum one-way travel time (1 h, 2 h or 3 h) and mode of transportation: walking only (W) or using a combination of motorized transportation, bicycling and walking (MBW). The boxes include median values and are bounded at upper and lower quartiles, with whiskers extending to 1.5 times the interquartile range and outliers shown as dots. Results are shown for all 9,208 communities in Malawi and for all 758 HCFs that provided ART in 2020. **a**, Boxplots showing the number of HCFs in the catchment area surrounding each community. **b**, Boxplots of the level of service at each HCF (the level of service is defined as the localized supply-to-demand ratio for ART in the catchment area around the HCF). **c**, Boxplots of the SAA index.

**Fig. 3:**
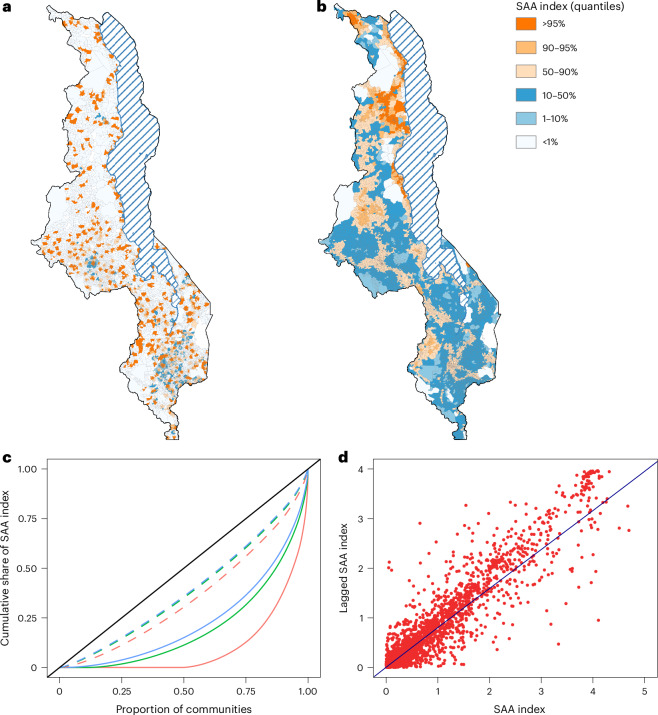
The SAA in Malawi. **a**, Map of the SAA index for a catchment size based on walking for a maximum of 1 h. The map shows values of the SAA index categorized into quantiles. **b**, Map of the SAA index for a catchment size based on using a combination of motorized transportation, bicycling and walking, and a maximum one-way travel time of 1 h. The map shows values of the SAA index categorized into quantiles. **c**, The Lorenz curves are shown for the six catchment sizes explored in the spatial sensitivity analysis; the diagonal line represents the line of equity in access to ART. Curves are shown for a maximum one-way travel time of 1 h (red), 2 h (green) and 3 h (blue) and mode of transportation: walking only (solid line) or using a combination of motorized transportation, bicycling and walking (dashed line). **d**, The Moran scatterplot shows the association between the value of the SAA index and its neighboring (spatially lagged) value. Results are for a catchment size based on using a combination of motorized transportation, bicycling and walking, and a maximum travel time of 1 h. The value of the Global Moran Index is 0.79; this indicates a very high degree of geographic clustering of communities with similar values of the SAA index.

**Extended Data Fig. 1:**
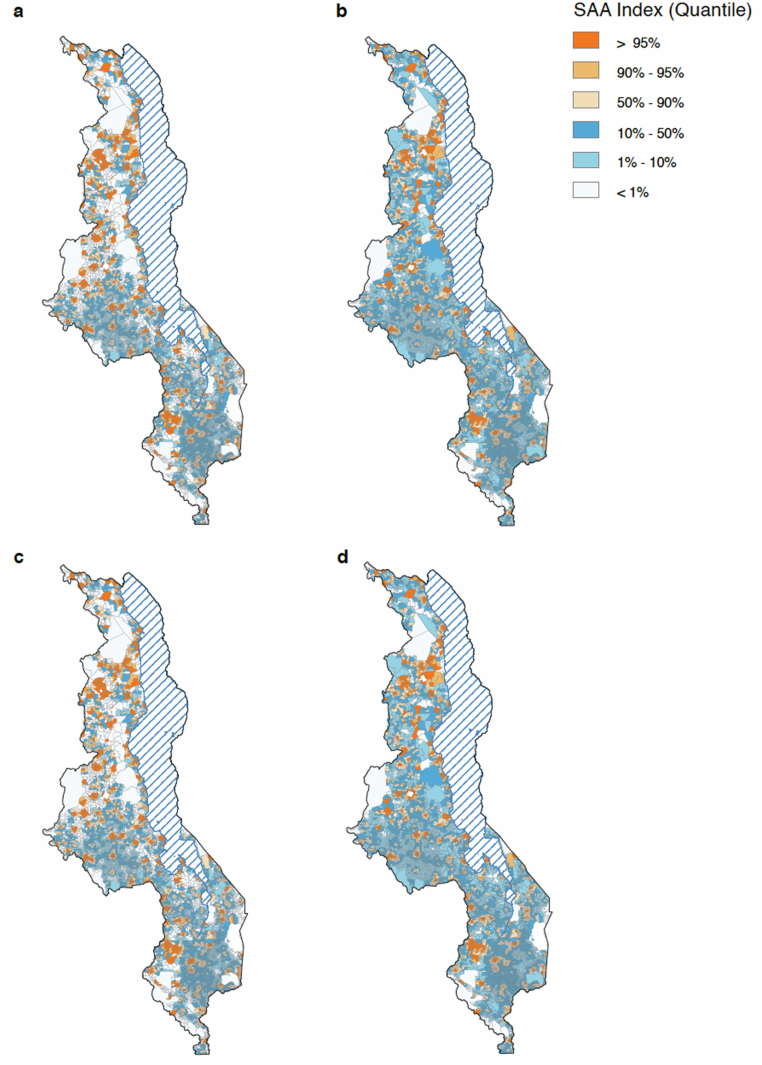
Maps of the SAA index in Malawi. **a–d,** Maps of the SAA index are shown in the form of quantiles, for four of the catchment sizes that are used in the spatial sensitivity analysis. Specifically, for catchment sizes based on: (**a**) walking for a maximum of 2 h; (**b**) walking for a maximum of 3 h; (**c**) using a combination of motorized transportation, biking, and walking for a maximum of 2 h; and (**d**) using a combination of motorized transportation, biking, and walking for a maximum of 3 h.

**Extended Data Fig. 2:**
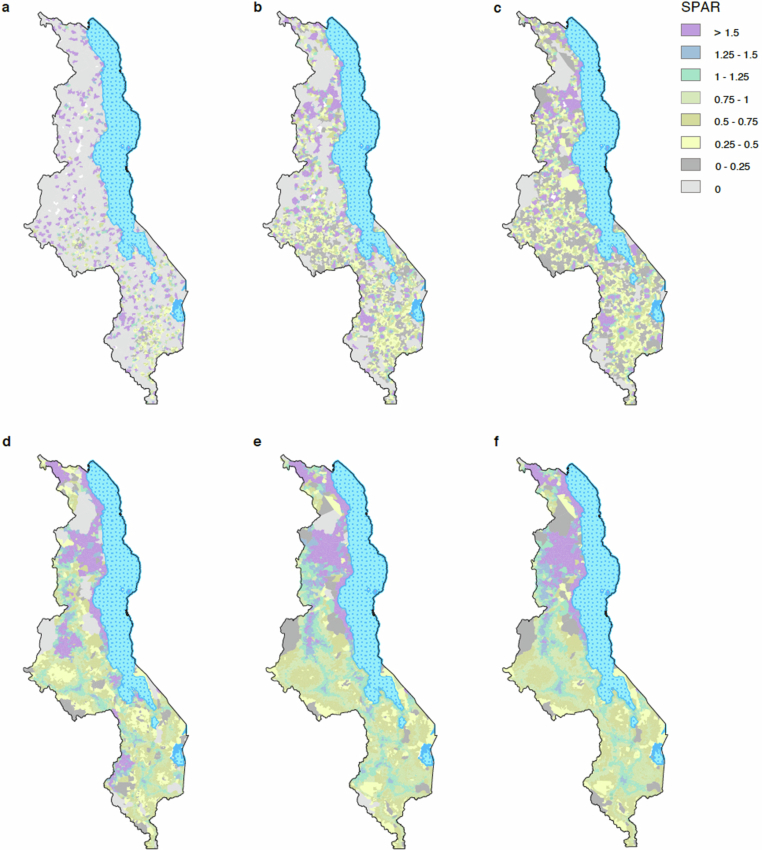
Maps of the SPAR in Malawi. The SPAR is a measure, for each community, of its accessibility to the available supply of ART relative to the national average. **a–f,** The maps show the SPAR for each of the six catchment sizes that are used in the spatial sensitivity analysis, where individuals: (**a**) walk for a maximum of 1 h; (**b**) walk for a maximum of 2 h; (**c**) walk for a maximum of 3 h; (**d**) use a combination of motorized transportation, biking, and walking for a maximum of 1 h; (**e**) use a combination of motorized transportation, biking, and walking for a maximum of 2 h; or (**f**) use a combination of motorized transportation, biking, and walking for a maximum of 3 h.

The 9,208 communities varied considerably (range: 0–332) in the number of HCFs in their catchment area (Fig. 2a and Extended Data Table 1). The 758 HCFs showed considerable variation in their localized supply-to-demand ratio for ART (Fig. 2b and Supplementary Table 1). Variation in these two factors resulted in substantial variation, among communities, in the value of their SAA index, that is, in their access to ART (Fig. 2c and Extended Data Table 2). Geographic variation in access is shown in Fig. 3a,b and Extended Data Fig. 1; access to ART in certain communities was substantially higher or lower than the national average (Extended Data Fig. 2 and Extended Data Table 2). For example, considering the catchment size based on walking for a maximum of 3 h, access to ART in some communities was ~36 times greater than the national average; however, some communities had no access.

On average, the number of HCFs in the catchment area around a community increases with catchment size (Fig. 2a); varying the maximum one-way travel time (from 1 h to 3 h) has less effect on the rate of increase than changing the mode of transportation. Changing travel speeds has a similar effect as changing the maximum one-way travel time (Extended Data Table 1 and Supplementary Table 2). The value of the supply-to-demand ratio for ART (Fig. 2b) and the SAA index (Fig. 2c) are relatively insensitive to catchment size, except for the smallest size. However, it is unlikely that this catchment size was the ‘true’ size because only ~60% of people living with HIV can reach an HCF within an hour of walking (Extended Data Table 1), whereas in 2020–2021, 86% of people living with HIV were known to be receiving ART at HCFs^11^.

### Lorenz curves and Gini coefficients

The results of our country-level equity evaluation of access to ART are shown in terms of Lorenz curves^26^ (Fig. 3c) and their corresponding Gini coefficients^27^ (Supplementary Table 3). These results reveal that the population’s lack of access to transportation had a substantial impact on generating inequity in access to ART in Malawi. For example, considering the largest catchment size based on a travel time of 3 h, the Gini coefficient increases from 0.21 (assuming individuals can use transportation) to 0.53 (assuming individuals have to walk). We identify a relationship between travel time (if walking) and inequity in access: the further that people living with HIV are able to walk (that is, the larger the catchment size), the lower the inequity. Taken together, our econometric results show that access to ART in Malawi in 2020 was highly inequitable: based on walking, the Gini coefficient was between 0.53 and 0.79.

### Identifying HIV treatment deserts

We found significant spatial autocorrelation in the values of the SAA index for all six catchment sizes: the Global Moran’s Index^28^ varies from 0.34 to 0.91 (*P* < 0.001; Fig. 3d and Supplementary Table 4). Therefore, regardless of catchment size, there is significant geographic clustering of communities with very similar values (either very high or very low) of the SAA index. These results reveal the existence of a new type of medical desert: an HIV treatment desert. We define HIV treatment deserts as areas where there is significant (assessed at *α* = 0.05) spatial clustering among communities, and all communities have very low values of the SAA index, that is, very low access to ART. These communities had significantly lower values of the SAA index than communities outside deserts (*P* < 0.001; Supplementary Table 5). Our results also reveal the existence of areas where there is significant spatial clustering, and communities have very high values of the SAA index: in these areas, ART is highly accessible. We subsequently refer to these clusters as clusters of abundance.

**Fig. 4:**
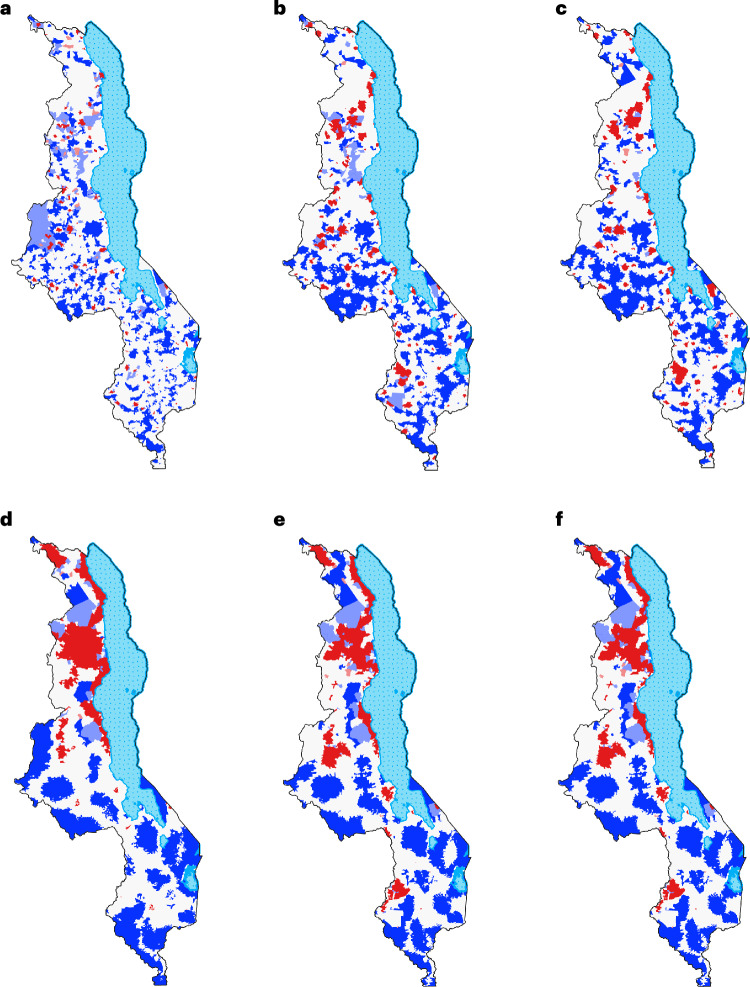
LISA cluster maps of the SAA index. LISA cluster maps show the localized spatial autocorrelation of the SAA index for communities throughout Malawi. Clusters are areas where all communities have a significantly (assessed at *α* = 0.05) lower SAA index (blue) or higher SAA index (red) than surrounding communities. The map also shows areas that are spatial outliers: areas where communities with a low SAA index (light blue) are surrounded by communities with a significantly higher SAA index or where communities with a high SAA index (pink) are surrounded by communities with a significantly lower SAA index. Areas that were neither part of clusters or spatial outliers are shown in light gray. Clusters in blue show the geographic location of HIV treatment deserts. Clusters in red show the geographic location of clusters of abundance. **a**–**f**, LISA cluster maps are shown for the six catchment sizes explored in the spatial sensitivity analysis, based on walking for a maximum of 1 h (**a**); walking for a maximum of 2 h (**b**); walking for a maximum of 3 h (**c**); using a combination of motorized transportation, bicycling and walking for a maximum of 1 h (**d**); using a combination of motorized transportation, bicycling and walking for a maximum of 2 h (**e**); and using a combination of motorized transportation, bicycling and walking for a maximum of 3 h (**f**).

The Local Index of Spatial Association (LISA)^29^ cluster maps show striking patterns in terms of the two distinct types of clusters: HIV treatment deserts and clusters of abundance (Fig. 4). The maps reveal the location and delimit the geographic boundaries of both types. HIV treatment deserts contain 15–23% of people living with HIV (16–27% of communities; Supplementary Tables 6 and 7). Clusters of abundance contain 4–13% of people living with HIV (5–11% of communities; Supplementary Tables 6 and 7). In each LISA cluster map, the spatial clustering that generates the HIV treatment deserts and the clusters of abundance contribute to the positive global spatial autocorrelation shown by the Global Moran’s Index. Areas that contain communities with high values of the SAA index and neighboring communities with low values, and areas that contain communities with low values of the SAA index and neighboring communities with high values, are spatial outliers. Essentially, no people living with HIV live in areas that are spatial outliers. The majority of communities (62–76%) are, with respect to their SAA index, randomly distributed: there is no evidence of a significant spatial association with neighboring communities.

Multiple HIV treatment deserts of varying sizes existed in Malawi in 2020 (Fig. 4). If people living with HIV have to walk to access ART—as catchment size increases—the number of deserts decreases from 168 to 74, but their size increases from 15% to 23% of people living with HIV (and from 16% to 27% of communities): there are fewer, but larger, deserts (Extended Data Table 3). If people living with HIV can use transportation, increasing catchment size has relatively little impact on the number of deserts (range: 43–46) or their size (range: 22–23% of people living with HIV, 25–26% of communities; Extended Data Table 3).

### Spatial uncertainty analysis

**Fig. 5:**
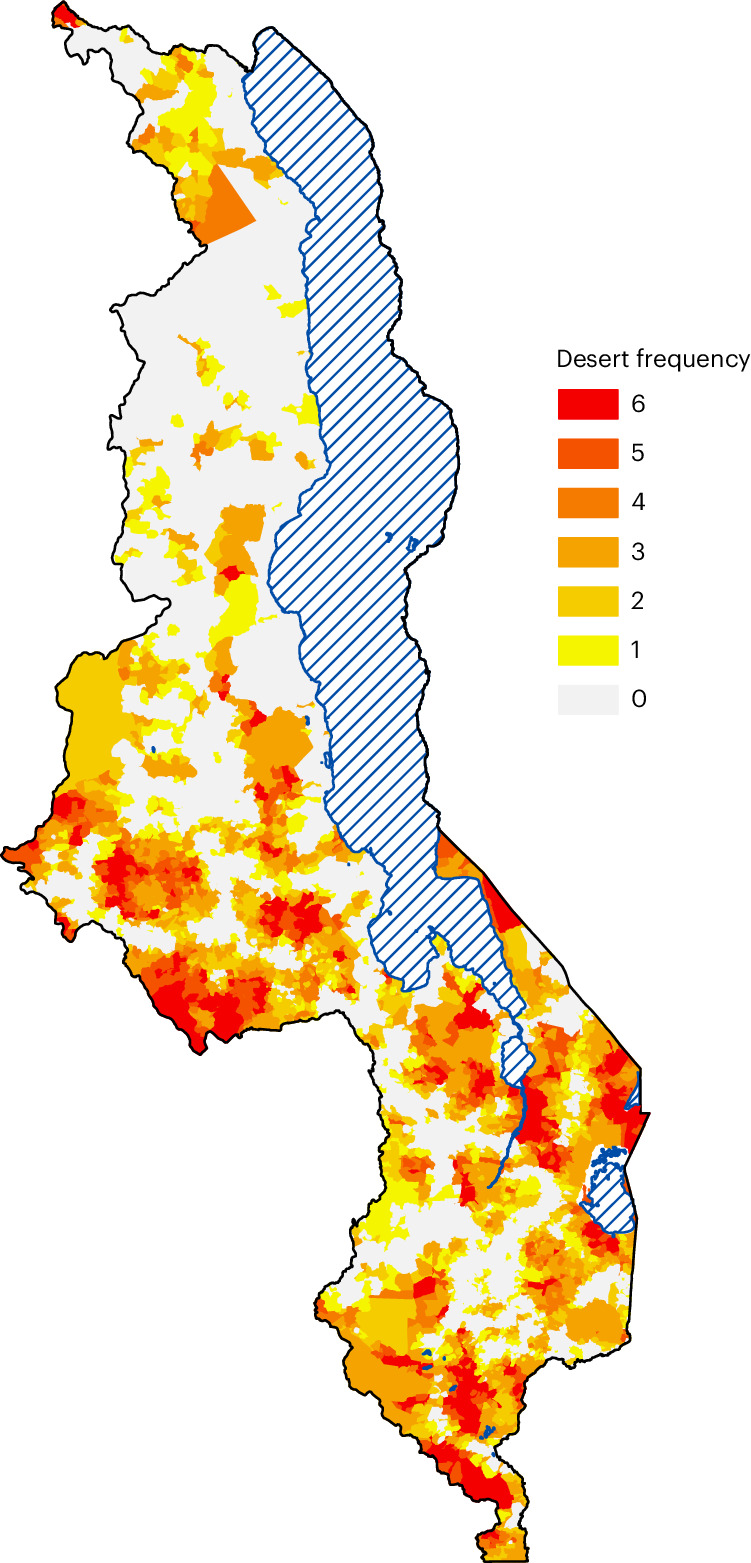
Heat map of HIV treatment deserts. These are the results of the spatial uncertainty analysis. The map shows the number of times a community is found in an HIV treatment desert, ranging from 0 (the community is never found in a desert) to 6 (the community is always found in a desert). Water bodies are shown with striped blue lines.

The results from our spatial uncertainty analysis are shown in the form of a heat map (Fig. 5). The map shows the number of times each of the 9,208 communities in Malawi is found in an HIV treatment desert: a value of 0 signifies that the community is never found in a desert, and a value of 6 signifies that the community is always found in a desert (that is, for every catchment size). The map shows that there are certain communities that are almost always found in deserts, regardless of assumptions about catchment size. This demonstrates the consistency of our results in identifying the existence and geographic location of HIV treatment deserts in Malawi.

### Characterizing HIV treatment deserts

To determine the extent to which communities in HIV treatment deserts were underserved with respect to HIV healthcare, we compared them with communities in the rest of Malawi. We compared the number of HCFs, the type of healthcare services these HCFs provided, the percentage of the national supply of ART these HCFs received and the number of people living with HIV (per 100) who received ART at these HCFs.

We first determined (based on geographic coordinates) which of the 758 HCFs that provided ART in 2020 (Fig. 1b) were inside and which were outside deserts. Our results show that deserts, regardless of catchment size, contained disproportionately fewer HCFs than areas outside deserts (Table 1). For example, if the catchment size was based on walking for up to 3 h, deserts only contained 7% of the HCFs providing ART despite containing 23% of the HIV-infected population.

**Table 1:** HIV healthcare resources available inside and outside HIV treatment deserts in 2020

Catchment	W, 1 h		W, 2 h		W, 3 h		MBW, 1 h		MBW, 2 h		MBW, 3 h	
Desert	In	Out	In	Out	In	Out	In	Out	In	Out	In	Out
PWH (%)	15	85	23	77	23	77	23	77	22	78	22	78
HCFs (%)	1	99	6	94	7	93	15	85	16	84	17	83
Secondary plus tertiary (%)	1	99	1	99	1	99	1	99	4	96	3	97
ART supply (%)	1	99	4	96	3	97	11	89	12	88	12	88
Number treated per 100 PWH	3	100	13	106	12	107	41	98	45	96	45	96

In 2020, 758 HCFs in Malawi were able to provide ART to 86% of people living with HIV. ‘PWH (%)’ is the percentage of people living with HIV that lived inside and outside deserts. ‘HCFs (%)’ is the percentage of HCFs that were located inside and outside deserts. ‘Secondary plus tertiary (%)’ is the percentage of all of the secondary and tertiary HCFs that were inside and outside deserts. ‘ART supply (%)’ is the percentage of the national supply of ART that the MoH provided to HCFs inside and outside deserts. ‘Number treated per 100 PWH’ is the number of people living with HIV that were treated with ART at HCFs per 100 people living with HIV inside and outside deserts. Results are shown for all six catchment sizes explored in the spatial sensitivity analysis: walking (W) or using a combination of motorized transportation, bicycling and walking (MBW) for a maximum one-way travel time of 1 h, 2 h or 3 h.

The distribution of the types of HCF that provided ART was very different inside and outside deserts (Supplementary Table 8). HCFs in Malawi provide three levels of care: primary, secondary and tertiary. Deserts only contained 1–4% of the HCFs that provided secondary or tertiary care, despite containing 15–23% of the HIV-infected population (Table 1). Healthcare services, in terms of both HIV prevention and treatment, were much more limited at HCFs providing primary care than at HCFs providing secondary and tertiary healthcare (Extended Data Table 4). Only secondary and tertiary HCFs provided treatment for tuberculosis and noncommunicable diseases (NCDs).

In 2020, the Ministry of Health (MoH) distributed a substantial supply of ART: enough to treat 86% of people living with HIV in the entire country^11^. We found that, regardless of catchment size, the geographic allocation of the national supply of ART was severely misaligned with respect to the geographic distribution of people living with HIV: deserts received a disproportionately smaller percentage of the national supply of ART than areas outside deserts (Table 1). For example, if the catchment size was based on walking for up to 3 h, HCFs in deserts only received 3% of the national supply of ART despite containing 23% of the HIV-infected population. In this case, HCFs only received enough ART to treat 12 per 100 people living with HIV, whereas HCFs outside deserts received enough ART to treat 107 per 100 people living with HIV (Table 1); this implies that some people living with HIV residing in treatment deserts traveled outside deserts for treatment. Access to transportation would have substantially decreased the geographic imbalance between deserts and areas outside deserts (Table 1).

## Discussion

Our study was motivated by the recently proposed UNAIDS human-rights-based approach for ending the pandemic by 2030^2^. We have developed a geospatial and geostatistical modeling framework, based on the concept of spatial accessibility and econometrics, and used it to conduct a country-level equity evaluation of access to ART. We have measured access to ART by developing a health services metric, the SAA index; this metric enables the identification of geographic gaps in health services. We have found that access to ART in Malawi in 2020 was extremely geographically inequitable at the national level and that this was, in part, owing to the population’s limited access to transportation. We have uncovered the existence of clusters of communities where ART was highly accessible and clusters of communities that had extremely low access to ART. These findings have led us to discover a new type of medical desert: the HIV treatment desert. We have found multiple, fairly large, HIV treatment deserts throughout Malawi: in these areas, there are substantial gaps in health services for HIV patients. Although there was enough ART in 2020–2021 to treat 86% of people living with HIV in the country^11^, there was an extreme geographic misalignment of healthcare resources with respect to need. This misalignment generated the HIV treatment deserts, as well as clusters of abundance where ART was highly accessible. Taken together, our results indicate that Malawi still has substantial challenges to meet to attain the 2030 goal of UNAIDS—achieving equity in access to HIV treatment.

Healthcare deserts create major and often complex problems for public health: they worsen health outcomes, increase healthcare costs and often compound economic burdens in socially vulnerable populations^3^. Many people living with HIV throughout SSA^30,31^ have comorbidities; in 2020 in Malawi, ~45% of people with active tuberculosis^32^ and ~8% of people diagnosed with NCDs^33^ were also infected with HIV. However, we found that people living with HIV who lived in deserts and chose not to (or were unable to) travel outside deserts to access healthcare would have been substantially less likely to receive treatment for HIV, TB or NCDs than people living with HIV in the rest of Malawi. Therefore, their health outcomes (on average and over the long term) would have been worse, and their life expectancy considerably shorter, than those of people living with HIV residing outside deserts. For example, the life expectancy of people living with HIV on ART is approximately equal to the life expectancy of persons without HIV^34^. In the absence of treatment, people living with HIV have a substantially reduced life expectancy: the average time from HIV infection to death is 8–13 years (ref. ^35^). Our results suggest that some residents of deserts chose to travel extremely long distances outside deserts to obtain healthcare; this does not negate the existence of HIV treatment deserts nor the importance of eliminating them. Healthcare deserts are defined based on the resources that residents of deserts can access within the desert’s geographic boundaries^3^. The current UNAIDS human-rights-based approach to eliminate HIV is based on achieving equity in access to HIV healthcare^2^.

The overall goal of public health is to provide equitable access to healthcare. Currently, many governments in SSA (South Africa, Kenya, Uganda and Malawi) use distance from HCFs as a measure of accessibility to healthcare and the percentage of the population that receives services as a measure of equity^25^. For example, the Government of Malawi has set a goal for 2030 of building or rehabilitating enough HCFs to ensure that 90% of their population lives within 5 km of an HCF^36^. The World Health Organization uses travel time to HCFs as a measure of access^37^; this is a more appropriate metric than distance, as it takes into account topography and road networks. The World Health Organization is currently constructing a database of the geographic location of HCFs in all 194 Member States^38^; their plan is to estimate travel times to HCFs and identify healthcare deserts. We^24^ and others^39–41^ have previously used travel time to HCFs to measure accessibility to healthcare. However, using travel time as a metric can result in overestimating accessibility (and hence underestimating the size or number of healthcare deserts) as the metric does not account for the supply-to-demand ratio for resources at HCFs. UNAIDS has called for the development of new spatial methods and metrics for measuring the accessibility of healthcare and equity in access^42^. Here we have presented a method and metric for measuring access and equity in access to ART. Our methodology could be used by any government in SSA to evaluate their level of equity in access to ART, to determine whether HIV treatment deserts exist in their country and to ascertain whether their healthcare resources are geographically aligned with need.

We have designed the mathematical model that we have presented here to calculate the accessibility of ART and HCFs. We have used it to evaluate equity in access to HIV healthcare at the national level and to reveal gaps in healthcare services. Our model differs from previous HIV models as it takes into consideration the geographic infrastructure of the healthcare system, travel time to access healthcare, transportation availability and the supply-to-demand ratio for ART at HCFs. Our modeling framework could help inform the design of geographically optimized HIV healthcare systems—specifically systems that provide equitable access to HIV healthcare. This approach could be developed by coupling geospatial optimization models with our FCA model and identifying systems that minimize countrywide differences in the SAA index. These coupled models could also be used to develop optimal solutions for providing and delivering HIV healthcare services and to design community-level ART allocation strategies that maximize equity in access. This is the subject of our current research with public health officials in Malawi. Finally, our modeling framework could be used to develop a new type of HIV transmission model that combines the geographic infrastructure of healthcare systems with the geographically varying transmission dynamics of HIV. The analysis of such models would lead to the identification of new types of epidemic control strategies that satisfy dual objectives: minimize HIV transmission and maximize equity in access to healthcare. These models could be used to investigate whether HIV treatment deserts serve as barriers to HIV elimination.

Communities with the lowest access to ART and HIV healthcare services live in HIV treatment deserts. These underserved communities are very likely to be in the most rural areas: areas that contain communities living in extreme poverty^43^. By identifying areas where there are gaps in health services for HIV patient care, our research provides actionable insights for health policy officials in Malawi. To minimize gaps, the accessibility of ART in deserts needs to increase. This could be accomplished by constructing new HCFs (which is a stated aim of the Government of Malawi^36^) or introducing and expanding non-facility-based delivery systems: for example, mobile deliveries^44,45^, drones^46^ or other non-facility-based systems^47^, either community based^48^ or patient centered^49,50^. Currently, non-facility-based delivery systems are only in their infancy or being piloted in Malawi^36,51,52^. All of these strategies would decrease inequalities in access to ART, shrink treatment deserts, increase treatment coverage in deserts and, potentially, reduce HIV transmission^53^. If deserts are not targeted, current geographic inequalities in access to ART in Malawi are likely to be exacerbated, and deserts will grow in size and number.

In 2020–2021, 14% of people living with HIV in Malawi were not on treatment^11^; our results suggest that a fairly high percentage of these people may be living in HIV treatment deserts and unaware of their status. Gaps in awareness of HIV status are an important driver of population-level HIV viremia in Malawi^9^. Therefore, we recommend instigating intensive targeted HIV-testing campaigns in HIV treatment deserts. These campaigns may lead to a higher-than-average yield of people living with HIV who are currently untreated. Testing campaigns may also potentially lead to the identification of HIV-negative individuals who have, because of where they live, a higher-than-average risk of infection; these individuals should be offered effective prevention modalities such as pre-exposure prophylaxis. However, to most effectively target prevention campaigns, prevention deserts, rather than treatment deserts, should be identified. Prevention and treatment deserts may or may not overlap. Pre-exposure prophylaxis deserts have recently been identified in the United States^54^.

Our study has several limitations. When using empirical Bayesian kriging (EBK)^55^ to generate the HIV prevalence map, we used geomasked cluster site locations from the 2020–2021 Malawi Population-Based HIV Impact Assessment (MPHIA2) survey^23^. The clusters were geomasked to ensure anonymization^56^; geomasking introduced location error and, hence, potential bias in our prevalence estimates. Our choice of the Worldpop dataset^57^ is also a potential limitation. There are several choices of population gridded datasets. We used the top-down constrained version of the 2020 WorldPop dataset that constrains the population to areas where settlements have been identified by high-resolution satellite data. We chose this version as it has been shown to be appropriate for modeling accessibility to healthcare^58^. In addition, our study is limited (as are all studies using FCA models) in that the exact size of catchment areas is unknown. However, our results are robust to catchment size; for all sizes, we identified substantial geographic inequity in access to ART and treatment deserts. Furthermore, we have found that, regardless of catchment size, some communities are always, or almost always, in deserts. Our study is potentially limited by data quality; there may be biases in the MPHIA2 data^23^ due to survey non-response. The MPHIA2 data that we have analyzed were collected in 2020–2021; treatment coverage is now slightly above 86%. Depending upon where coverage has increased, geographic inequalities in access to ART may have decreased or increased.

Taken together, our results show that there was substantial geographic misalignment in resources for HIV healthcare in Malawi in 2020 and that this led to the emergence of HIV treatment deserts. Based on human rights, deserts need targeting with an increased supply of ART to redress current inequalities in the provision of HIV healthcare. However, these may not be the most efficient strategies for reducing incidence because equity and efficiency can be in opposition^59^. Many other countries in SSA such as Lesotho, Eswatini and Zambia have similar characteristics to Malawi with respect to the geographic distribution of their healthcare systems and the geographic variation in their HIV epidemics^39,60^. Therefore, it is possible that many of these countries will also contain HIV treatment deserts. The modeling approach that we have used for HIV may be applicable to other regions in Africa to identify geographic inequalities in access to medicines and vaccines for other highly prevalent communicable diseases and, more importantly, to begin to determine how to redress these health inequities.

## Methods

### The balanced FCA model and the SAA index

To conduct our study, we used the balanced FCA (bFCA) model developed previously^22^ and used in other studies^20,61,62^. Conceptually, an FCA model computes the ratio of supply to demand within a catchment area centered at each supplier’s location, and then ‘floats’ these catchment areas over population centers to determine the allocation of the available resources to each of the demand sites. Catchment areas are delimited by specifying a maximum one-way travel time between the supplier’s location and the demand site. The bFCA model has an important advantage over the other types of FCA model as it corrects for issues of inflation of demand and service levels and takes competition among supply sites into account^22^. The bFCA model, as do all FCA models, produces an estimate of the spatial accessibility of a resource.

We used the bFCA model to estimate the SAA in Malawi, that is, to estimate, for each community in Malawi, their access to ART. The SAA reflects the geographic distribution of the HCFs that provide ART, the geographic distribution of the available supply of ART among HCFs, the geographic distribution of communities with people living with HIV, the mobility of the population (as specified by travel time to an HCF and mode of transportation) and the behavioral phenomenon of distance decay: the probability of using an HCF decreases as the time needed to travel to the HCF increases^63,64^. For a variety of reasons (for example, concern about being stigmatized), people living with HIV may choose not to use their nearest HCF; this behavior is referred to as bypass behavior and has been observed in SSA^65–67^. The bFCA model allows bypass behavior by letting people living with HIV use any of the HCFs that lie within their community’s catchment area.

The bFCA model is specified by five equations. For our application of the bFCA model, we specify communities as demand sites and HCFs as supply sites. The model includes *i* communities (*i* ∈ {1,…, *N*}) and *j* HCFs (*j* ∈ {1,…, *J*}). Equation (1) calculates the demand for ART at each HCF in the country; demand is specified in terms of the number of people living with HIV. The demand at each HCF depends upon how many communities are in its catchment area, how many people living with HIV each of these communities contain, the travel time from the HCF to each community, the mode of transportation used and the behavioral phenomenon of distance decay. It is defined by:

1 $${D}_{j}=\mathop{\sum }\limits_{{{i}}={{1}}}^{{{N}}}{{{P}}}_{{{i}}}{{{W}}}_{{{ij}}}^{\,{{i}}}$$

where the demand (*D*_*j*_) at HCF *j* is the sum of the number of people living with HIV (*P*_*i*_) in community *i*, weighted by the probability (${W}_{{ij}}^{\,i}$) that people living with HIV from community *i* use HCF *j*. ${W}_{{ij}}^{\,i}$ is a standardized impedance weight. People living with HIV from community *i* can use HCF *j* if community *i* lies within the catchment area of HCF *j*.

Impedance weights (*W*_*ij*_) provide a measure of the difficulty of moving from community *i* to HCF *j* given a specified mode of transportation. They are estimated by using a function *f*(∙) that depends on the travel time *t*_*ij*_ between community *i* and HCF *j*, using a specified mode of transportation. *f*(∙) is modeled with a decreasing function to represent the behavioral phenomenon of distance decay^63,64^. HCFs cannot be used by a community if they are outside the community’s catchment area. By evaluating *f*(∙) for all travel times *t*_*ij*_, impedance weights ${W}_{{ij}}=f\left({t}_{{ij}}\right)$ are obtained. The impedance weights are then standardized:

2 $${{{W}}}_{{{ij}}}^{\,{{i}}}={{{W}}}_{{{ij}}}/\mathop{\sum }\limits_{{{j}}}^{{{J}}}{{{W}}}_{{{ij}}}\;{\rm{such}}\; {\rm{that}}\mathop{\sum }\limits_{{{j}}}^{{{J}}}{{{W}}}_{{{ij}}}^{\,{{i}}}={{1}}$$

Equation 3 calculates the level of service (*L*_*j*_) at each HCF *j* in the country. The level of service at an HCF is a measure of the localized supply-to-demand ratio in the catchment area of that HCF. It is calculated by dividing the supply at the HCF by the localized demand at that HCF:

3 $${{{L}}}_{{{j}}}=\frac{{{{S}}}_{{{j}}}}{{{{D}}}_{{{j}}}}=\frac{{{{S}}}_{{{j}}}}{{\sum }_{{{i}}={\bf{1}}}^{{{N}}}{{{P}}}_{{{i}}}{{{W}}}_{{{ij}}}^{{{i}}}}$$

The supply *S*_*j*_ of each HCF *j* is defined to be the maximum quarterly number of people living with HIV treated at HCF *j* during the year.

Equation (4) calculates the SAA index for community *i* (SAA_*i*_). SAA_*i*_ is the weighted sum of the level of service at all of the HCFs that are contained within the catchment area of community *i*:

4 $${{\rm{SAA}}}_{{{i}}}=\mathop{\sum }\limits_{{{j}}={{1}}}^{{{J}}}{{{L}}}_{{{j}}}{{{W}}}_{{{ij}}}^{\,{{j}}}$$

Here the standardized impedance weight (${W}_{{ij}}^{\,j}$) is the probability that HCF *j* can be used by people living with HIV in community *i*; it is calculated as follows:

5 $${{{W}}}_{{{ij}}}^{\,{{j}}}={{{W}}}_{{{ij}}}/\mathop{\sum }\limits_{{{i}}}^{{{N}}}{{{W}}}_{{{ij}}}\;{\rm{such}}\;{\rm{that}}\mathop{\sum }\limits_{{{i}}}^{{{N}}}{{{W}}}_{{{ij}}}^{\,{{j}}}={{1}}$$

The standardized impedance weights produce the ‘balance’ in the model by preventing inflated demand and service levels, which occur in other types of FCA models^22^. For example, without these weights, there could be multiple communities with high probabilities of using the same HCF at levels that are not commensurate with the ART supply available at that HCF.

### The spatial potential accessibility ratio

The spatial potential accessibility ratio (SPAR) is a measure of a community’s accessibility to the available supply of healthcare resources relative to the national average^68^. For example, if a community has a SPAR of 0.5, then its accessibility to ART is 50% lower than average. A good score is a value of SPAR > 1; the higher the value, the better the accessibility to ART relative to the average. A bad score is a value of SPAR < 1; the lower the value, the worse the accessibility relative to the national average.

### Parameterization

To parameterize the model, we needed to know, for 2020, (1) the geographic location of every HCF that provided ART, (2) the supply of ART at each HCF, (3) the geographic location of every community in Malawi, (4) the number of people living with HIV in each community (demand) and (5) the standardized impedance weights. We programmed the model in R (v.4.1.2)^69^.

#### The geographic location of every HCF that provided ART

Each of the 758 HCFs that provided ART in 2020 was geolocated at the geographic coordinates (latitude and longitude) obtained from the master list provided by Malawi’s MoH.

#### The supply of ART at each HCF

Malawi’s government-funded national healthcare system is free for all Malawians at the point of delivery. The supply of ART (*S*_*j*_) at each HCF *j* was estimated from 2020 data provided by Malawi’s MoH. Malawi used a centralized ART distribution system that was based on push dynamics: (1) all HCFs that provided ART were consulted quarterly by the MoH as to how much ART they needed for the next 3 months, (2) they were allocated the amount they requested and (3) they distributed all of the ART that they received. Supply data were validated each quarter. Malawi uses multi-month scripting for ART: prescriptions are typically for 3 months. The distribution system ensured that all people living with HIV who requested ART in 2020 received treatment; there were no stock outs, and HCFs were not underutilized (that is, they did not have a supply of ART that was not utilized). Based on this distribution system, we defined the total supply of ART that was provided in 2020 as the maximum number of people living with HIV that were treated in 2020. We estimated the supply at each HCF by calculating the maximum quarterly number of people living with HIV (aged 15 or older) that were treated, at that specific HCF, in any one of the four quarters in 2020.

#### The geographic location of every community in Malawi

Each community was geolocated at the population-weighted centroid of their enumeration area^70^. The population was specified in terms of the number of people living with HIV in the community.

#### The number of people living with HIV in each community (demand)

**Extended Data Fig. 3:**
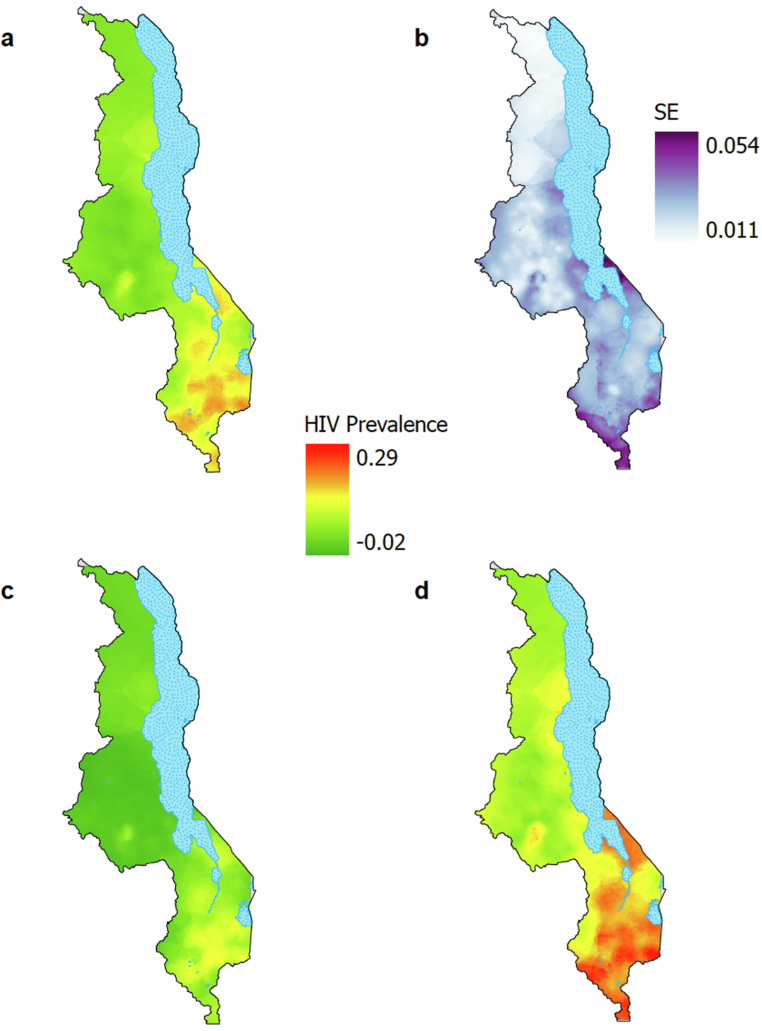
HIV prevalence map, standard error map, and 95% confidence interval maps. **a**, HIV prevalence map. The map was created by using EBK to spatially interpolate cluster-level HIV prevalence estimates calculated using data from MPHIA2. **b**, Standard error (SE) map. **c**, Map showing 95% lower bound for HIV prevalence. **d**, Map showing 95% upper bound for HIV prevalence. Maps (a), (c), and (d) are plotted on the same scale. There are several places in central Malawi where the 95% lower bound falls below zero. This is to be expected—despite the SEs being fairly small (mean 0.03)—because HIV prevalence is as low as 0.02 in some areas. Validation statistics for the EBK model are provided in Supplementary Table 9.

We estimated the demand for ART in each community in terms of the total number of people living with HIV they contained. To estimate these numbers, we first constructed an HIV prevalence map for people living with HIV aged 15 or older (Extended Data Fig. 3a; maps of the corresponding 95% confidence intervals and standard errors are shown in Extended Data Fig. 3b–d). The prevalence map was based on HIV-testing data collected in MPHIA2^23^. The MPHIA2 survey collected blood samples from a representative sample of the population of Malawi in 2020–2021^11^. These data were collected between January 2020 and April 2021; the majority were collected in 2020. The survey used a two-stage cluster sampling design. All individuals were nested within georeferenced survey clusters; the clusters were geomasked to ensure anonymization^56^. The individual-level data on HIV testing (*n* = 22,662) were aggregated at the cluster level.

**Extended Data Fig. 4:**
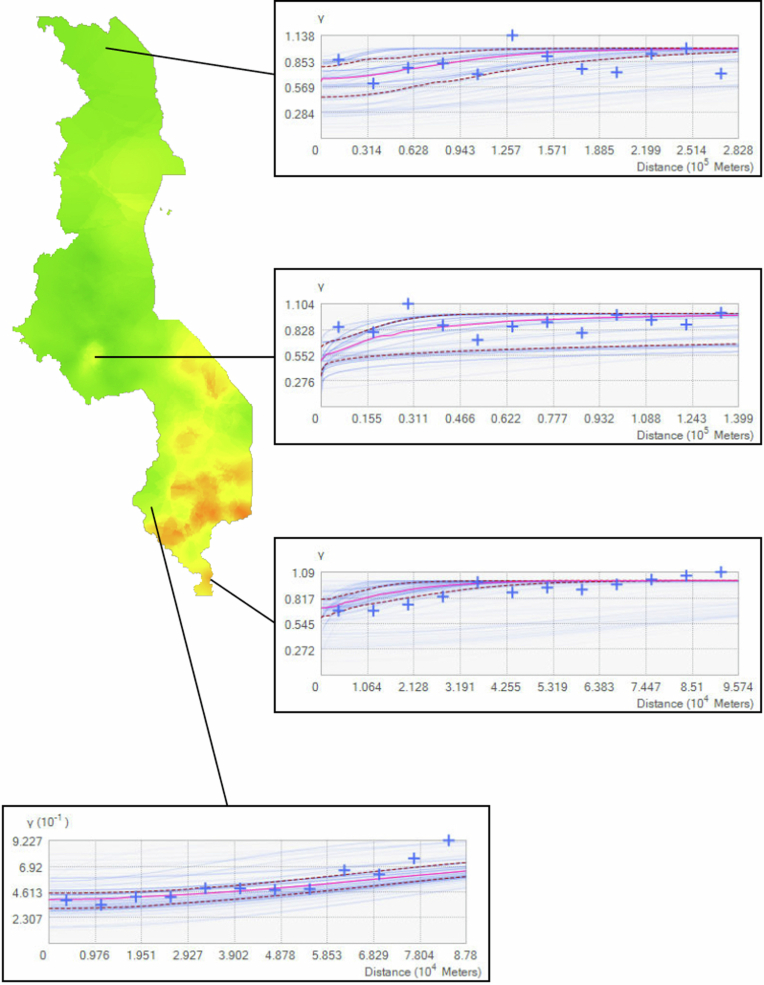
Semivariograms from EBK model. The background map shows the HIV prevalence map, as in Extended Data Fig. 3a. The insets provide a visualization of the distribution of semivariograms used in the EBK model, and how the distribution varies geographically. The spectrum of semivariograms is shown with light blue lines, the median of the distribution is shown by a solid pink line, the 25th and 75th percentiles by dashed red lines, and the empirical semivariances by the blue crosses.

We created the HIV prevalence map by using EBK^55^ to spatially interpolate the cluster-level HIV prevalence estimates calculated from the MPHIA2 data. EBK is a geostatistical technique for spatial interpolation; it uses a function (in our case, a *K*-Bessel function) to model the empirical semivariogram. The semivariogram reflects the degree of spatial correlation in the data. EBK accounts for the error in estimating the semivariogram by deriving a distribution of empirical semivariograms at each location. A geographic visualization of the distribution of semivariograms at four different locations is shown in Extended Data Fig. 4. We used cross-validation to assess how well the EBK model was able to predict values at locations where HIV prevalence data had not been collected. Cross-validation metrics are shown in Supplementary Table 9.

**Extended Data Fig. 5:**
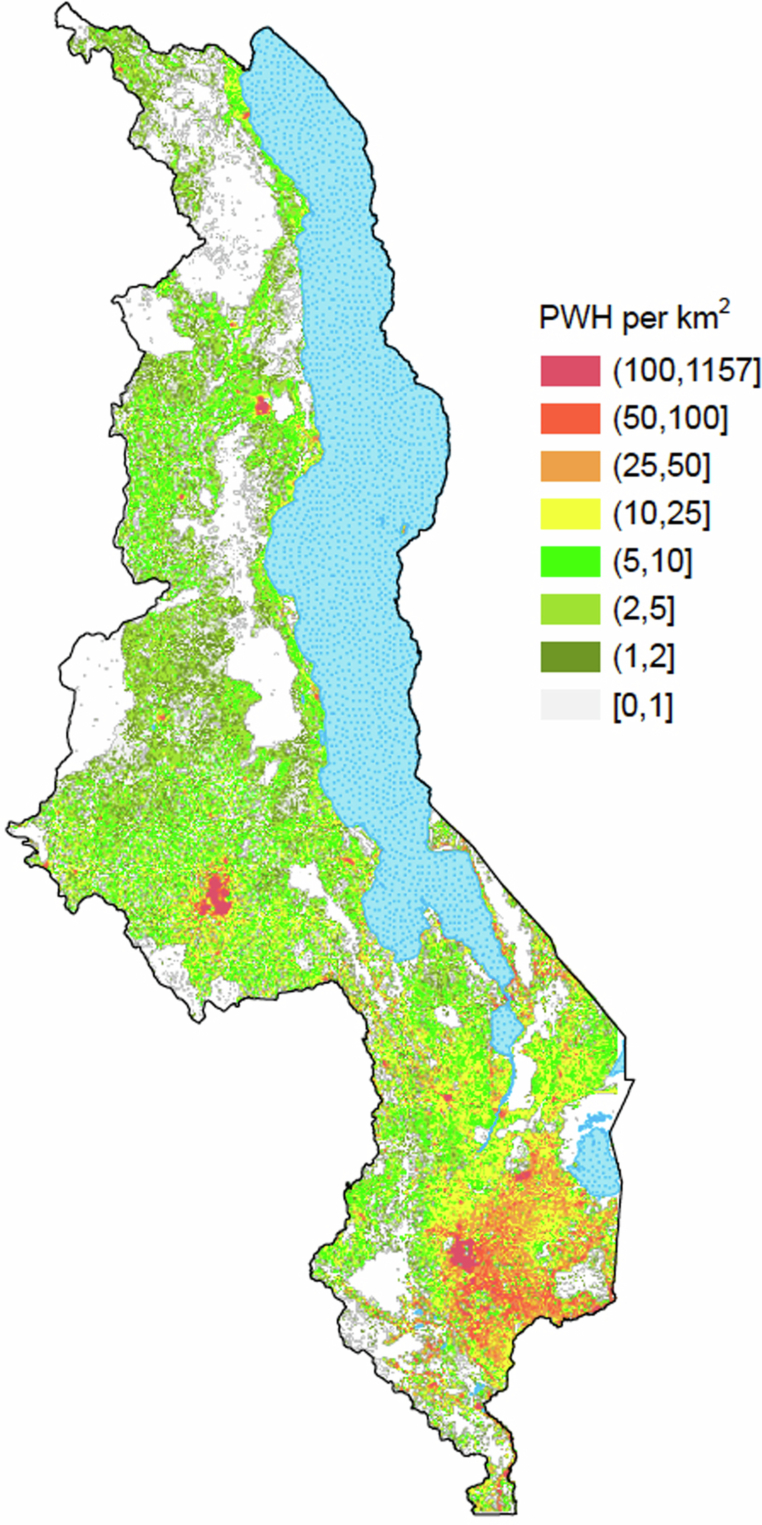
DoI map of Malawi. The DoI map was constructed at a spatial resolution of 100 m by 100 m, and is presented here aggregated to a resolution of 1 km by 1 km.

After constructing the HIV prevalence map, we combined it (using raster multiplication) with the gridded raster dataset (for 15 years and older) of the 2020 WorldPop data^57^ for Malawi; this produced a density of infection (DoI) map (Extended Data Fig. 5). WorldPop data are gridded data of population density at a resolution of 100 m by 100 m; data are updated annually to reflect UNAIDS-predicted urban–rural growth rates. We used the top-down constrained version of the 2020 WorldPop dataset. The DoI map was constructed at a spatial resolution of 100 m by 100 m. We then used ArcGIS to partition the DoI map into the 9,208 communities in Malawi and estimated the number of people living with HIV in each community. The total number of people living with HIV in community *i* is *P*_*i*_.

#### The standardized impedance weights

To calculate the standardized impedance weights, we first calculated an origin–destination (OD) matrix of travel times. In the OD matrix, the columns represent HCFs and the rows represent communities; the coefficients of the matrix specify the travel time *t*_*ij*_ between every community *i* and every HCF *j*, based on a specified mode of transportation. As in all FCA models, all travel times begin from the population-weighted centroid of the community’s enumeration area. To calculate travel times, we used an impedance map^24^. This map is essentially a three-dimensional representation of Malawi; it includes data on topography^71^, land cover^72^, rivers and other water bodies^72^, and road networks^73^. The map provides estimates of the time needed for an average individual to traverse each square kilometer of Malawi, using a specified mode of transportation. We calculated this map using AccessMod (v.5)^37^, geospatial data files^70–73^ and travel speeds for several modes of transportation (Supplementary Table 10). Previous studies have used Google Maps Platform Application Programming Interfaces to estimate travel times to HCFs in Africa^74,75^. We used the platform to estimate the average travel time needed to travel 1 km in Malawi (for each type of road in our study; Supplementary Table 10); we then calculated the reciprocal of this value to obtain the average travel speed (in kilometers per hour). Using these travel speeds and the impedance map, we calculated the travel times between all HCFs and all communities. As there are 758 HCFs and 9,208 communities in Malawi, there are ~7 million coefficients in the OD matrix.

**Extended Data Fig. 6:**
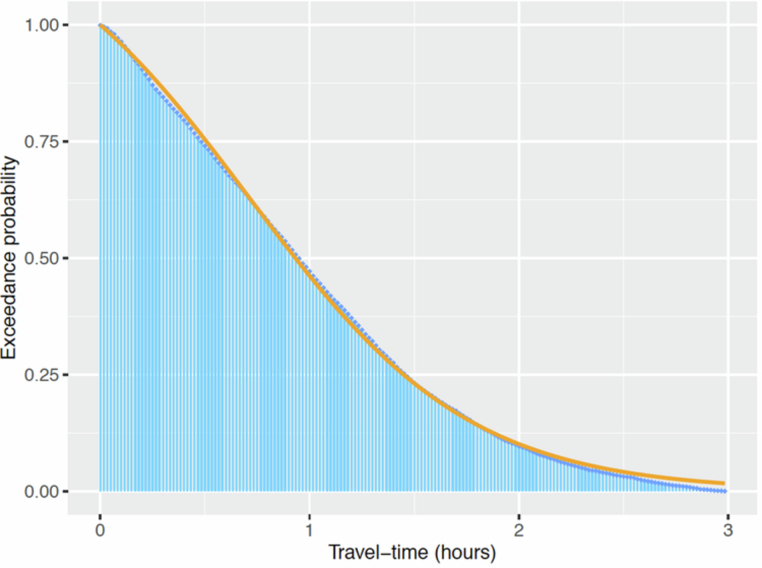
Distance decay function. The distance decay function is shown in orange. It shows the relationship between travel time and the ‘exceedance probability’, that is, the cumulative proportion of communities that can reach a HCF in a certain travel time (or longer). The distance decay function was estimated by fitting a logistic function through data (blue) on the travel times between each community and the nearest HCF (Residual Sum of Squares, RSS = 0.0019).

We then used the OD matrix and a distance decay function *f*(∙) to calculate the impedance matrix. *f*(∙) was estimated by using a data-based methodology that was designed to estimate a distance decay function for an FCA model^76^. The distance decay function fitted to the data is shown in Extended Data Fig. 6. Using this function enabled us to operationalize the phenomenon of distance decay: the further individuals have to travel to reach an HCF, the less likely they are to visit the HCF; this phenomenon has frequently been found to occur in SSA^63,64^. We calculated the coefficients for the impedance matrix by evaluating *f*(∙) for all travel times in the OD matrix (that is, we calculated *f*(*t*_*ij*_)). Notably, the majority of the coefficients were zero, as people living with HIV in any given community are not able to reach the majority of HCFs in the country within their specified maximum one-way travel time. The impedance matrix was then row standardized and column standardized, as in equations (2) and (5), respectively, to ensure that the population was allocated proportionally to the HCFs. The resulting matrices contain the standarized impedance weights ${{{W}}}_{{{ij}}}^{{{i}}}$ and ${{{W}}}_{{{ij}}}^{\,{{j}}}$ for the model.

We calculated standardized impedance weights based on each mode of transportation: walking only or a combination of motorized transportation, bicycling and walking.

### Spatial sensitivity analysis

To conduct the spatial sensitivity analysis, we varied catchment size; the size was defined by setting catchment boundaries. Boundaries were set by constructing an impedance map of Malawi^24^, specifying a maximum one-way travel time between supply sites (HCFs) and demand sites (communities), and stipulating a mode of transportation.

We varied two factors that delimit catchment boundaries: the maximum time that people living with HIV spend traveling (one way) to HCFs to receive their medications and the type of transportation that they use to reach HCFs. The 2020–2021 MPHIA2 data indicate that 44% of people living with HIV on ART spent less than 1 h traveling to an HCF, 37% spent 1–2 h and 19% spent more than 2 h (ref. ^23^). We used three values to specify the maximum one-way travel time: 1 h, 2 h or 3 h. The MPHIA2 survey also collected data on the ownership of different types of transportation. Only 2% of households owned cars or trucks, 4% owned motorbikes or scooters, and 34% owned bicycles^23^. Therefore, we modeled two modes of transportation: the slowest possible (only walking) and the fastest possible. The fastest possible mode was based on using a combination of three types of transportation: motorized transportation, bicycling and walking. The type of transportation used depends upon the type of road or track that is traveled on (Supplementary Table 10). By crossing the two factors (the maximum one-way travel time and the mode of transportation), we examined six catchment sizes in the spatial sensitivity analysis. The longer the maximum travel time and/or the faster the mode of transportation, the larger the catchment. The smallest catchment size was based on walking one way for a maximum of 1 h. The largest catchment size was based on using a combination of the three types of transportation and traveling one way for a maximum of 3 h.

### Varying travel speeds

We conducted an analysis to investigate the impact of varying travel speeds on the geographic accessibility of HCFs. Following published methods^41,77^, we examined slower and faster travel speeds relative to the baseline travel speeds that we used in our spatial sensitivity analysis (Supplementary Table 10): specifically ±20% of the baseline travel speeds. We considered two modes of transportation: walking or using a combination of motorized transportation, bicycling and walking, with a maximum one-way travel time of 2 h.

### Model verification

In all bFCA models, the sum of the level of service (equation (3)) for all of the HCFs should equal the sum of the SAA index (equation (4)) for all of the communities. We verified the bFCA model that we used in our analysis by checking, for all six catchment sizes in the spatial sensitivity analysis, that this relationship held.

### Calculating Lorenz curves and Gini coefficients

We conducted a country-level equity evaluation of access to ART in Malawi in 2020 by using econometrics to calculate and visualize an overall summary measure of the degree of inequity in access. The Lorenz curve^26^ and the Gini coefficient^27^ are metrics developed over a century ago to quantify economic inequities at the national level. If the income distribution in a population is perfectly equal, the Lorenz curve is a diagonal line; the further the curve is from the diagonal, the greater the inequity. The Gini coefficient measures the area between the Lorenz curve and the line of absolute equality, and is expressed as a percentage of the maximum area under the line. Thus, a Gini coefficient of zero represents perfect equity, while a value of one implies complete inequity. Both the Lorenz curve and Gini coefficient have previously been used to measure health inequities^78,79^.

First, we computed a Gini coefficient based on each of the six catchment sizes explored in the spatial sensitivity analysis. We then constructed the six corresponding Lorenz curves. Lorenz curves were constructed by computing the cumulative distribution function of the SAA index.

### Geostatistical clustering analysis

To determine whether there was significant spatial clustering in communities based on their SAA index, we calculated the Global Moran’s Index^28^. This index measures the strength of the spatial autocorrelation between neighboring communities and varies from −1 to +1; negative values signify dispersion and positive values signify clustering of communities with similar values of the SAA index. For the SAA indices generated, based on the six catchment sizes explored in the spatial sensitivity analysis, we calculated the Global Moran’s Index. We then calculated the LISA statistic^29^ and plotted country-level LISA cluster maps.

### Spatial uncertainty analysis

To determine the robustness of our results in identifying the existence and geographic location of HIV treatment deserts, we conducted a spatial uncertainty analysis. To conduct this analysis, we determined, for each of the six catchment sizes that were explored in the spatial sensitivity analysis, which communities occurred in deserts and then plotted the results in the form of a heat map of Malawi. The heat map shows the number of times a community is found in an HIV treatment desert: a value of 0 signifies that the community living in that specific location is never found in a treatment desert and a value of 6 signifies that the community living in that specific location is always found (that is, for every catchment size) in a treatment desert.

### Calculating the numbers treated per 100 people living with HIV

We calculated, using data from the MoH, the number of people living with HIV (per 100 people living with HIV) who were treated with ART at HCFs inside deserts by (1) summing the number of people living with HIV who were treated with ART at every HCF that was in a desert, (2) summing the number of people living with HIV in every community that was in a desert and (3) dividing (1) by (2) and multiplying by a hundred. We made the same calculation for the number of people living with HIV (per 100 people living with HIV) who were treated with ART at HCFs outside deserts.

### Statistical analyses

Statistical analyses were performed in R (v.4.1.2)^69^ and GeoDa (v.1.22.0.4)^80^. Summary numbers and statistics are presented as means unless otherwise indicated. Two-sample *t*-tests were used to compare the mean SAA index inside and outside deserts; significance was assessed at *α* = 0.05. The LISA cluster maps were created in GeoDa; clusters and spatial outliers were assessed using two-tailed tests at a significance level of *α* = 0.05.

### Ethics and inclusion statement

This project was initiated by researchers at the University of California, Los Angeles, in collaboration with researchers and medical doctors from Partners in Health in Malawi, and the Ministry of Health of the Government of Malawi. As such, this paper includes authors from many backgrounds throughout the international scientific community. Roles and responsibilities were agreed upon before the research was conducted. From the early stages of the project, all team members collaborated on data ownership and study design. Our study is focused on Malawi; therefore, in-country experts were team members. These team members played a critical role in providing detailed knowledge of Malawi’s medical system with a focus on HIV treatment. All team members are co-authors of this paper. We have cited local and regional research that is relevant to our study. As our study has focused only on modeling, capacity building has not been discussed.

This study involves secondary analysis of data that were collected from previous studies. The MPHIA2 data that we have used are publicly available; during its original collection, the PHIA study protocols were reviewed and approved by in-country ethics and regulatory bodies. The Malawi ART supply data were provided to us in the form of aggregated, de-identified HCF-level data.

### Reporting summary

Further information on research design is available in the Nature Portfolio Reporting Summary linked to this article.

## Online content

Any methods, additional references, Nature Portfolio reporting summaries, source data, extended data, supplementary information, acknowledgements, peer review information; details of author contributions and competing interests; and statements of data and code availability are available at 10.1038/s41591-025-03561-6.

## Supplementary information

Supplementary Information Supplementary Tables 1–10.

Reporting Summary

## Figures and Tables

**Extended Data Table 1:**
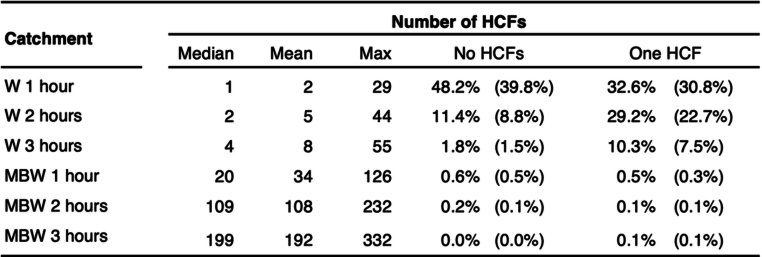
The number of healthcare facilities (HCFs) within a community’s catchment area

Results are shown for the six catchment sizes explored in the spatial sensitivity analysis. Catchment sizes are based on varying the maximum one-way travel time (one, two, or three hours), and mode of transportation: walking only (W), or using a combination of motorized transportation, bicycling and walking (MBW). The travel speeds used in this analysis (that is, baseline travel speeds) are shown in Supplementary Table 10. Results show the percentage of communities (and, in parenthesis, the percentage of people living with HIV) that cannot access any HCFs in the maximum one-way travel time (this is labeled as: No HCFs), and the percentage of communities (and, in parenthesis, the percentage of people living with HIV) that can only access one HCF in the maximum one-way travel time (this is labeled as: One HCF).

**Extended Data Table 2:**
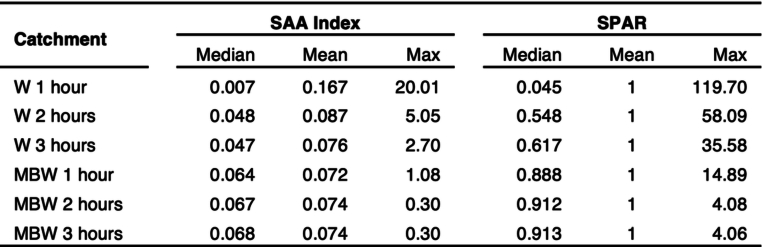
The Spatial Accessibility of ART (SAA) index and the Spatial Potential Accessibility Ratio (SPAR)

Results are shown for the six catchment sizes explored in the spatial sensitivity analysis. Catchment sizes are based on varying the maximum one-way travel time (one, two, or three hours), and mode of transportation: walking only (W), or using a combination of motorized transportation, bicycling and walking (MBW).

**Extended Data Table 3:**
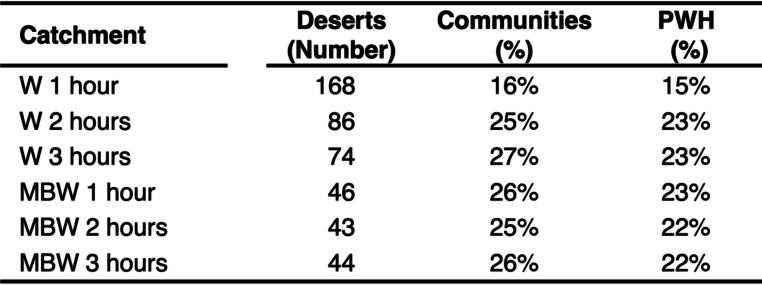
Number and size of HIV treatment deserts

Results show, for the six catchment sizes explored in the spatial sensitivity analysis, the number of HIV treatment deserts and percentage of communities and people living with HIV that they contain. Catchment sizes are based on varying the maximum one-way travel time (one, two, or three hours), and mode of transportation: walking only (W), or using a combination of motorized transportation, bicycling and walking (MBW).

**Extended Data Table 4:**
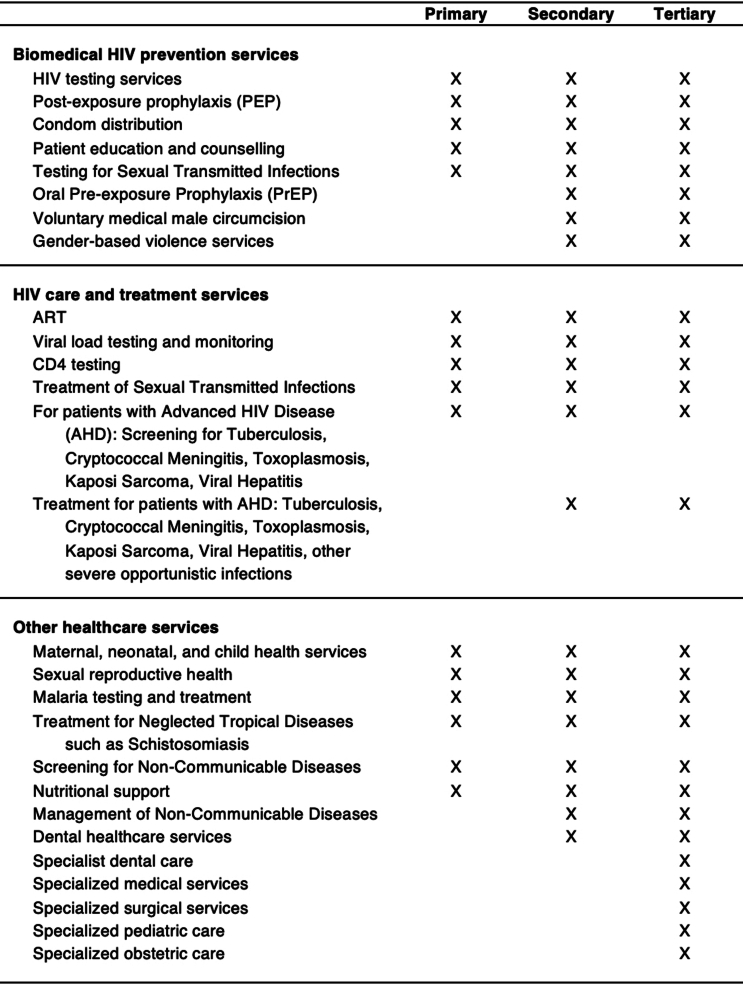
Healthcare services available at all three levels of care in Malawi

## Data Availability

PHIA data are freely available for registered users at the PHIA project website: https://phia-data.icap.columbia.edu/. WorldPop’s spatial demographic data are freely available at https://www.worldpop.org/. Malawi HIV clinic geolocations and ART supply data were obtained from the MoH in Malawi and cannot be provided for reasons of confidentiality.

## Code availability

The code used to carry out these analyses is available via GitHub at https://github.com/joanponce90/bFCA-model-ART-MWI.
