# Supplementary material for: Extreme geographic misalignment of healthcare resources and HIV treatment deserts in Malawi
Source: Nat Med. 2025 Mar 14;31(5):1474–83. doi: 10.1038/s41591-025-03561-6 (PMC12092277; doi:10.1038/s41591-025-03561-6)
Supplement: Supplementary file 1 — Supplementary Tables 1–10. [file 41591_2025_3561_MOESM1_ESM.pdf]

nature medicine

Supplementary information https://doi.org/10.1038/s41591-025-03561-6

Extreme geographic misalignment of healthcare resources and HIV treatment deserts in Malawi

In the format provided by the authors and unedited

Table of contents Page

S1	**Supplementary Table 1**. The level of service at each of the 758 HCFs that provided ART.	2
S2	**Supplementary Table 2**. The effect of varying travel speeds on the number of HCFs in a community's catchment area.	2
S3	**Supplementary Table 3**. Gini coefficient (*G*) for each of the six catchment sizes explored in the spatial sensitivity analysis.	3
S4	**Supplementary Table 4**. Global Moran's Index (*I*) for each of the six catchment sizes explored in the spatial sensitivity analysis.	3
S5	**Supplementary Table 5**. The values of the SAA Index inside, and outside, deserts.	3
S6	**Supplementary Table 6**. Results from the LISA cluster maps showing the percentage of people with HIV in each cluster or spatial outlier.	4
S7	**Supplementary Table 7**. Results from the LISA cluster maps showing the percentage of communities in each cluster or spatial outlier.	4
S8	**Supplementary Table 8**. Type of HCF found inside, and outside, of HIV treatment deserts.	5
S9	**Supplementary Table 9**. Validation statistics for the Empirical Bayesian Kriging (EBK) model that was used to construct the HIV prevalence map shown in Extended Data Figure 3a.	6
S10	**Supplementary Table 10**. Baseline travel speeds specified by terrain and mode of transportation.	6

Catchment	Level of service		
	Median	Mean	Max
W 1 hour	1.248	2.021	30.90
W 2 hours	0.651	1.052	19.02
W 3 hours	0.569	0.917	14.12
MBW 1 hour	0.583	0.875	13.37
MBW 2 hours	0.575	0.888	13.45
MBW 3 hours	0.571	0.894	13.52

Supplementary Table 1. The level of service at each of the 758 HCFs that provided ART. The level of service at a HCF is defined as the localized supply to demand ratio for ART in that HCF's catchment area. Results are shown for the six catchment sizes explored in the spatial sensitivity analysis. Catchment sizes are based on varying the maximum one-way travel-time (one, two, or three hours), and mode of transportation: walking only (W), or using a combination of motorized transportation, bicycling and walking (MBW).

Transportation & travel speed	Number of HCFs						
	Median	Mean	Max	No HCFs		One HCF	
W lowest	1	3	40	21.6%	(16.4%)	37.0%	(30.3%)
W baseline	2	5	44	11.4%	(8.8%)	29.2%	(22.7%)
W highest	3	6	49	6.0%	(4.6%)	20.1%	(14.8%)
MBW lowest	60	75	191	0.3%	(0.2%)	0.1%	(0.1%)
MBW baseline	109	108	232	0.2%	(0.1%)	0.1%	(0.1%)
MBW highest	129	127	251	0.1%	(0.0%)	0.1%	(0.1%)

Supplementary Table 2. The effect of varying travel speeds on the number of HCFs in a community's catchment area. Results from an analysis examining the effect of varying travel speeds on the number of HCFs that can be accessed by a community within a maximum one-way travel-time of 2 hours, using a specific mode of transportation: walking (W) or using a combination of motorized transportation, bicycling, and walking (MBW). Results are shown for three travel speeds: baseline (given in Supplementary Table 10), lowest (20% below baseline), and highest (20% above baseline). Results show the percentage of communities (and, in parenthesis, the percentage of people with HIV) that cannot access any HCFs in the maximum one-way travel-time (this is labeled as: No HCFs), and the percentage of communities (and, in parenthesis, the percentage of people with HIV) that can only access one HCF in the maximum one-way travel-time (this is labeled as: One HCF).

Catchment	*G*
W 1 hour	0.79
W 2 hours	0.60
W 3 hours	0.53
MBW 1 hour	0.30
MBW 2 hours	0.23
MBW 3 hours	0.21

Supplementary Table 3. Gini coefficient (*G*) for each of the six catchment sizes explored in the spatial sensitivity analysis. Catchment sizes are based on varying the maximum one-way travel-time (one, two, or three hours), and mode of transportation: walking only (W), or using a combination of motorized transportation, bicycling and walking (MBW).

Catchment	*I*
W 1 hour	0.34
W 2 hours	0.75
W 3 hours	0.79
MBW 1 hour	0.79
MBW 2 hours	0.90
MBW 3 hours	0.91

Supplementary Table 4. Global Moran's Index (*I*) for each of the six catchment sizes explored in the spatial sensitivity analysis. Catchment sizes are based on varying the maximum one-way travel-time (one, two, or three hours), and mode of transportation: walking only (W), or using a combination of motorized transportation, bicycling and walking (MBW).

Desert:	**Inside**		**Outside**		**Significance**	
Catchment	Mean	IQR	Mean	IQR	t	*P*
W 1 hour	0.001	(0.000,0.000)	0.198	(0.000,0.213)	29.7	<.001
W 2 hours	0.012	(0.000,0.020)	0.112	(0.039,0.132)	47.7	<.001
W 3 hours	0.015	(0.007,0.021)	0.098	(0.041,0.114)	57.5	<.001
MBW 1 hour	0.036	(0.029,0.045)	0.085	(0.060,0.091)	73.6	<.001
MBW 2 hours	0.045	(0.039,0.052)	0.083	(0.064,0.090)	80.0	<.001
MBW 3 hours	0.046	(0.042,0.053)	0.083	(0.065,0.092)	81.0	<.001

Supplementary Table 5. The values of the SAA Index inside, and outside, deserts. The Table shows summary statistics: mean, and interquartile range (IQR). Two-sample t-tests were used to compare the mean SAA Index inside, and outside, deserts; significance was assessed at $\alpha=0.05$. Results are shown for the six catchment sizes explored in the spatial sensitivity analysis. Catchment sizes are based on varying the maximum one-way travel-time (one, two, or three hours), and mode of transportation: walking only (W), or using a combination of motorized transportation, bicycling and walking (MBW).

Classification	W 1 hour	W 2 hours	W 3 hours	MBW 1 hour	MBW 2 hours	MBW 3 hours
Not significant	78%	66%	65%	69%	68%	64%
Treatment desert	15%	23%	23%	23%	22%	22%
Cluster of abundance	4%	10%	11%	8%	10%	13%
Low-high spatial outlier	2%	0%	0%	0%	0%	0%
High-low spatial outlier	0%	0%	0%	0%	0%	0%

Supplementary Table 6. Results from the LISA cluster maps showing the percentage of people with HIV in each cluster or spatial outlier. A treatment desert is a cluster of communities with very low values of the SAA Index. A cluster of abundance is an area where all communities have very high values of the SAA Index. A low-high spatial outlier is an area that contains communities with low values of the SAA Index and neighboring communities with high values. A high-low spatial outlier is an area that contains communities with high values of the SAA Index and neighboring communities with low values. Results are shown for the six catchment sizes explored in the spatial sensitivity analysis. Catchment sizes are based on varying the maximum one-way travel-time (one, two, or three hours), and mode of transportation: walking only (W), or using a combination of motorized transportation, bicycling and walking (MBW). Columns may not sum to 100% due to rounding.

Classification	W 1 hour	W 2 hours	W 3 hours	MBW 1 hour	MBW 2 hours	MBW 3 hours
Not significant	76%	64%	62%	63%	65%	63%
Treatment desert	16%	25%	27%	26%	25%	26%
Cluster of abundance	5%	10%	11%	10%	10%	11%
Low-high spatial outlier	2%	1%	0%	1%	0%	0%
High-low spatial outlier	0%	0%	0%	0%	0%	0%

Supplementary Table 7. Results from the LISA cluster maps showing the percentage of communities in each cluster or spatial outlier. A treatment desert is a cluster of communities with very low values of the SAA Index. A cluster of abundance is an area where all communities have very high values of the SAA Index. A low-high spatial outlier is an area that contains communities with low values of the SAA Index and neighboring communities with high values. A high-low spatial outlier is an area that contains communities with high values of the SAA Index and neighboring communities with low values. Results are shown for the six catchment sizes explored in the spatial sensitivity analysis. Catchment sizes are based on varying the maximum one-way travel-time (one, two, or three hours), and mode of transportation: walking only (W), or using a combination of motorized transportation, bicycling and walking (MBW). Columns may not sum to 100% due to rounding.

	Catchment:	W 1 hour		W 2 hours		W 3 hours		MBW 1 hour		MBW 2 hours		MBW 3 hours	
	Desert:	Inside	Outside	Inside	Outside	Inside	Outside	Inside	Outside	Inside	Outside	Inside	Outside
HCF type	Primary	88%	75%	98%	73%	96%	73%	98%	71%	94%	71%	95%	71%
	Secondary	0%	12%	0%	13%	0%	13%	2%	14%	3%	14%	2%	14%
	Tertiary	13%	13%	2%	14%	4%	14%	0%	16%	2%	15%	2%	15%

Supplementary Table 8. Type of HCF found inside, and outside, of HIV treatment deserts. HCFs are classified by the level of healthcare (primary, secondary, or tertiary) that they provide. Results are shown for the six catchment sizes explored in the spatial sensitivity analysis. Catchment sizes are based on varying the maximum one-way travel-time (one, two, or three hours), and mode of transportation: walking only (W), or using a combination of motorized transportation, bicycling and walking (MBW). Columns may not sum to 100% due to rounding.

EBK cross-validation metrics	Value
Mean Error	0.00
Root Mean Square	0.06
Mean Standardized	0.00
RMS–Standardized	0.99
Ave Standard Error	0.06
Average CRPS	0.03
Inside 90% Interval	90.8%
Inside 95% Interval	95.0%

Supplementary Table 9. Validation statistics for the Empirical Bayesian Kriging (EBK) model that was used to construct the HIV prevalence map shown in Extended Data Figure 3a. EBK was conducted using a K-Bessel semivariogram model. The EBK model performs well across all cross-validation metrics.

	Drive, bike and walk		Walk only	
Terrain	Speed (km/h)	Mode	Speed (km/h)	Mode
Shrubland	4	WALKING	4	WALKING
Herbaceous vegetation	4	WALKING	4	WALKING
Cropland	4	WALKING	4	WALKING
Built-up areas	15	BICYCLING	4	WALKING
Bare sparse vegetation	4	WALKING	4	WALKING
Herbaceous wetland	4	WALKING	4	WALKING
Evergreen broadleaf forests	4	WALKING	4	WALKING
Deciduous broadleaf forests	4	WALKING	4	WALKING
Trunk road	60	MOTORIZED	4	WALKING
Primary road	50	MOTORIZED	4	WALKING
Secondary road	40	MOTORIZED	4	WALKING
Tertiary road	30	MOTORIZED	4	WALKING
Residential/unclassified road	30	MOTORIZED	4	WALKING
Service road	25	MOTORIZED	4	WALKING
Path/footway/track	4	WALKING	4	WALKING
Bridleway/cycleway	15	BICYCLING	4	WALKING
Pedestrian street	4	WALKING	4	WALKING

Supplementary Table 10. Baseline travel speeds specified by terrain and mode of transportation. These travel speeds were used for the six catchment sizes explored in the spatial sensitivity analysis.
