# Supplementary material for: Extreme geographic misalignment of healthcare resources and HIV treatment deserts in Malawi
Source: Nat Med. 2025 Mar 14;31(5):1474–83. doi: 10.1038/s41591-025-03561-6 (PMC12092277; doi:10.1038/s41591-025-03561-6)
Supplement: Supplementary file 2 — Reporting Summary [file 41591_2025_3561_MOESM2_ESM.pdf]

nature portfolio

Corresponding author(s): Sally Blower

Last updated by author(s): Jan 15, 2025

Reporting Summary

Nature Portfolio wishes to improve the reproducibility of the work that we publish. This form provides structure for consistency and transparency in reporting. For further information on Nature Portfolio policies, see our Editorial Policies and the Editorial Policy Checklist.

Statistics

For all statistical analyses, confirm that the following items are present in the figure legend, table legend, main text, or Methods section.

n/a	Confirmed	
☒	☐	The exact sample size (*n*) for each experimental group/condition, given as a discrete number and unit of measurement
☒	☐	A statement on whether measurements were taken from distinct samples or whether the same sample was measured repeatedly
☐	☒	The statistical test(s) used AND whether they are one- or two-sided<br>*Only common tests should be described solely by name; describe more complex techniques in the Methods section.*
☒	☐	A description of all covariates tested
☒	☐	A description of any assumptions or corrections, such as tests of normality and adjustment for multiple comparisons
☐	☒	A full description of the statistical parameters including central tendency (e.g. means) or other basic estimates (e.g. regression coefficient) AND variation (e.g. standard deviation) or associated estimates of uncertainty (e.g. confidence intervals)
☒	☐	For null hypothesis testing, the test statistic (e.g. *F*, *t*, *r*) with confidence intervals, effect sizes, degrees of freedom and *P* value noted<br>*Give P values as exact values whenever suitable.*
☒	☐	For Bayesian analysis, information on the choice of priors and Markov chain Monte Carlo settings
☒	☐	For hierarchical and complex designs, identification of the appropriate level for tests and full reporting of outcomes
☒	☐	Estimates of effect sizes (e.g. Cohen's *d*, Pearson's *r*), indicating how they were calculated

Our web collection on statistics for biologists contains articles on many of the points above.

Software and code

Policy information about availability of computer code

Data collection	Not applicable as no data were collected.
Data analysis	We used Accessmod (v.5) to create an impedance map and calculate travel times. The balanced Floating Catchment Area (bFCA) model was programmed in R (v.4.1.2) to analyze biometric and treatment data. Statistical analyses of the outputs from the bFCA mode were performed in R (v.4.1.2) and GeoDa (v.1.22.0.4). Code used to carry out these analyses is available from a Github repository: https://github.com/joanponce90/bFCA-model-ART-MWI

For manuscripts utilizing custom algorithms or software that are central to the research but not yet described in published literature, software must be made available to editors and reviewers. We strongly encourage code deposition in a community repository (e.g. GitHub). See the Nature Portfolio guidelines for submitting code & software for further information.

Data

Policy information about availability of data

All manuscripts must include a data availability statement. This statement should provide the following information, where applicable:

- Accession codes, unique identifiers, or web links for publicly available datasets
- A description of any restrictions on data availability
- For clinical datasets or third party data, please ensure that the statement adheres to our policy

PHIA data are freely available for registered users at the PHIA project website: https://phia-data.icap.columbia.edu/. WorldPop's spatial demographic data are freely

available at: https://www.worldpop.org/. Malawi HIV clinic geolocations and ART supply data were obtained from the MoH in Malawi, and cannot be provided for reasons of confidentiality.

Research involving human participants, their data, or biological material

Policy information about studies with human participants or human data. See also policy information about sex, gender (identity/presentation), and sexual orientation and race, ethnicity and racism.

Reporting on sex and gender	We analyzed data that had been collected in previous studies. Our analyses were at the population-level regarding access to HIV treatment, and did not differentiate on the basis of sex or gender. Furthermore, while the MPHIA data can be disaggregated by sex (self-reported), the ART regimen data (that is necessary to run the model) cannot be disaggregated by sex or gender. For this reason, sex- or gender-specific analyses could not be considered in the study design.
Reporting on race, ethnicity, or other socially relevant groupings	We analyzed data that had been collected in previous studies. We only used data from adults (ages 15 and up). Our analyses were at the population-level regarding access to HIV treatment, and did not differentiate on the basis of race, ethnicity, or other social groupings.
Population characteristics	This is a secondary data analysis using data from the entire adult population of Malawi. All men and women ages 15 and up were included.
Recruitment	We did not recruit any participants.
Ethics oversight	Not applicable. Our analysis was a modeling analysis using data collected in previous studies.

Note that full information on the approval of the study protocol must also be provided in the manuscript.

Field-specific reporting

Please select the one below that is the best fit for your research. If you are not sure, read the appropriate sections before making your selection.

☒ Life sciences ☐ Behavioural & social sciences ☐ Ecological, evolutionary & environmental sciences

For a reference copy of the document with all sections, see nature.com/documents/nr-reporting-summary-flat.pdf

Life sciences study design

All studies must disclose on these points even when the disclosure is negative.

Sample size	We did not do any sampling. This is a modeling study, for which we perform a secondary data analysis on several datasets that are freely available online. The data for the entire adult (ages 15 and up) population of Malawi in 2020 (N=10,897,547) comes from WorldPop. The HIV-testing data (N = 22,662) used to estimate prevalence, was previously collected as part of the MPHIA2 survey.
Data exclusions	No data were excluded from the study.
Replication	We did not conduct any experiments. We analyzed previously collected data. Custom code was verified by two authors. Repeat calculations were successful in verifying accuracy.
Randomization	Not applicable, as we did not conduct an experimental study. We conducted a modeling study based on previously collected data.
Blinding	Not applicable, as we did not conduct an experimental study. We conducted a modeling study based on previously collected data.

Reporting for specific materials, systems and methods

We require information from authors about some types of materials, experimental systems and methods used in many studies. Here, indicate whether each material, system or method listed is relevant to your study. If you are not sure if a list item applies to your research, read the appropriate section before selecting a response.

Materials & experimental systems

n/a	Involved in the study
☒	☐ Antibodies
☒	☐ Eukaryotic cell lines
☒	☐ Palaeontology and archaeology
☒	☐ Animals and other organisms
☒	☐ Clinical data
☒	☐ Dual use research of concern
☒	☐ Plants

Methods

n/a	Involved in the study
☒	☐ ChIP-seq
☒	☐ Flow cytometry
☒	☐ MRI-based neuroimaging

Plants

Seed stocks	N/A
Novel plant genotypes	N/A
Authentication	N/A
